# RNA-seq-based analysis of drug-resistant *Salmonella enterica* serovar Typhimurium selected *in vivo* and *in vitro*

**DOI:** 10.1371/journal.pone.0175234

**Published:** 2017-04-05

**Authors:** Lin Li, Xingyang Dai, Ying Wang, Yanfei Yang, Xia Zhao, Lei Wang, Minghua Zeng

**Affiliations:** Pharmacology and Toxicology Laboratory, College of Animal Science and Technology, Anhui Agricultural University, Hefei, P. R. China; Cornell University, UNITED STATES

## Abstract

The aim of this study was to characterize the mechanism of fluoroquinolone (FQ) resistance in *Salmonella* Typhimurium. We established the *Caenorhabditis elegans*–*Salmonella* Typhimurium model to select for ciprofloxacin resistance in *Salmonella* Typhimurium colonizing *C*. *elegans*, generating the resistant strains TN4. Gradient doses of ciprofloxacin were used to generate the resistant strain TW4 *in vitro*. RNA sequencing was used to establish the whole-transcriptome profile of three strains of *Salmonella* Typhimurium. The gene expression patterns of resistant strains TN4 and TW4 differed from those of the parental strain. In TN4, 2,277 genes were differentially expressed (1,833 upregulated and 444 downregulated) relative to the parental strain, and in TW4, 3,464 genes were differentially expressed (3,433 upregulated and 31 downregulated). Among these differentially expressed genes, 28 were associated with drug resistance and 26 were associated with the two-component systems in the two resistant strains. Seven different pathways were significantly sffected in two strains. Efflux pump overexpression was identified as one of the main mechanisms underlying FQ resistance in the two resistant strains. TW4 differentially expressed more efflux pump genes than TN4 and most of these genes were more strongly expressed than in TN4. However, expression of the efflux pump repressor gene and the mar operon was downregulated in TN4 but not in TW4. Two-component systems are also important in drug resistance. Our findings provide an important basis for further studies of the complex network that regulate FQ resistance in *Salmonella*.

## Introduction

*Salmonella* species are common zoonotic pathogens of the family Enterobacteriaceae that infect humans and animals, causing gastroenteritis, wound infections, bacteremia and other diseases. Fluoroquinolones (FQs) are a class of primary antibiotics used in the treatment of *Salmonella* infections, and their inappropriate use has led to enhanced FQ resistance in *Salmonella* species [1]. Therefore, it is important to better understand the mechanisms underlying the increased resistance of *Salmonella* spp. to controll the generation and propagation of FQ resistance.

The mechanism of FQ resistance in susceptible bacteria are typically investigate *in vitro*. However, this approach does not accurately reflect the complex biological environment *in vivo*. No recent reports have describ the selection of bacterial FQ resistance *in vivo*. *Caenorhabditis elegans* is a model organism in which the mechanisms of pathogen activation, the infection process, the immune response, and regulation of life activities are very similar to those in mammals [2–5]. Stable *in vivo Salmonella* Typhimurium infections can be established in *Caenorhabditis elegans* [6,7], so we used this *in vivo* model to select for FQ resistance in this study.

Transcriptomics is a technique used to investigate changes in RNA expression under specific circumstances [8]. Sequencing techniques are widely used in basic research, clinical diagnostics, drug development, and other fields to investigate overall patterns of gene structure and function, and there are numerous reports of bacterial transcriptome studies.

In this study, *in vitro*-cultured *Salmonella* Typhimurium was used to colonize *C*. *elegans*, which was then exposed to ciprofloxacin to produce a resistant bacterial strain *in vivo*. The parental *Salmonella* strain and those showing selective drug resistance, generated *in vitro* and *in vivo*, were analyzed with cDNA sequencing to determine variation at the transcriptome level and provide important data resources for further investigation of the molecular mechanisms and metabolic pathways associated with FQ resistance in *Salmonella* Typhimurium.

## Materials and methods

### Bacterial and nematode strains

*Salmonella* Typhimurium strain ATCC 13311 (parental strain T0) and *Escherichia coli* strain ATCC 25922 were grown on Mueller–Hinton broth (MHB) medium at 37°C. Wild-type *C*. *elegans* strain N2 was maintained by using standard methods [9], and was grown on nematode growth medium (NGM) containing *E*. *coli* OP50, with incubation at 20°C.

### Ethics statement

The study was carried out in laboratory of Anhui Agricultural University and Shanghai biotechnology corporation, no specific permissions were required for these locations. The field studies did not involve endangered or protected species. All bacterial and nematode strains including selected resistant strains in this study were preserved in our laboratory.

### Construction of recombinant *Salmonella* Typhimurium–green fluorescent protein (GFP) strain

Competent cells were prepared from *Salmonella* Typhimurium ATCC 13311 with the chemical (calcium chloride) method. The competent cells were then transformed with the plasmid pET30a-GFP using electroporation. The recombinant strain was cultured on Luria-Bertani agar containing kanamycin (100 μg/ml), and then selected strain was grown for PCR identification. Expression of the identified strain weas induced with isopropyl β-D-thiogalactoside and observed under a fluorescence microscope. A recombinant strain was used to colonize the nematodes, which were then washed three times with M9 buffer containing sodium azide, and placed on a slide for observation under a fluorescence microscope.

### Selected of ciprofloxacin-resistant strain *in vivo*

Nematode synchronization [10]. Adult nematodes from NGM were washed with M9 buffer in a sterile tube and with 2.8 ml of control M9 buffer. Sodium hypochlorite (0.8 ml, 7%) and sodium hydroxide solution (0.4 ml, 5 mol/l) were then added and mixed well, and the mixture was allowed to stand for 10 min. The nematodes were then centrifuged for 1 min at 2,000 × *g*, washed three times with sterile M9 buffer, and transferred to M9 buffer containing egg on a sterile plate, for incubation at 20°C. After 24 h, the nematodes grown to the L1-stage were transferred to NGM containing *E*. *coli* OP50 and incubated at 20°C.

Bacterial colonization and selection *in vivo*. The L1-syage nematodes had grown to L4-stage after 48–52h. They were washed three times with M9 buffer and collected. The appropriate amount of nematodes was transferred to plates containing *Salmonella* Typhimurium and cultured for 12 h at 20°C. The infected nematodes were then washed from the plate with M9 buffer. The nematodes (1,000–2,000) were transferred to 50 ml of M9 buffer containing ciprofloxacin (0.004 μg/ml) and grown in a shaking incubator at 20°C. After 24–36 h, the nematodes were centrifuged for 1 min at 2,000 × *g* to remove the M9 buffer and washed three times with M9 buffer containing 1 mmol/l sodium azide. Ten nematodes were transferred to a sterile tube containing 400 mg of quartz sand and 1 ml sterile water, and ruptured by vortexing for 2–3 min. The diluted supernatant was spread onto *Salmonella* Typhimurium chromogenic medium and incubated at 37°C.

Generation and identification of drug-resistant strain. Typical colonies was observed on the chromogenic medium, indicating that the strain adapt to the concentration of ciprofloxacin in the M9 buffer. Single colonies were selected to prepare infection plates. We increased the drug concentrations in M9 buffer two-fold and repeated the experimental procedure until a resistant bacteria was obtained (ciprofloxacin minimum inhibitory concentration [MIC] = 4 μg/ml). At the same time, a control was established in which no drug was added during the nematode incubation process. The resistant strain was cultured five times in MHB with no drug, and its drug sensitivity was evaluated to determine the stability of the drug-resistance phenotype. The resistant strain selected *in vivo* was designated ‘TN4’.

### Selection of ciprofloxacin-resistant strains *in vitro*

A resistant strain was selected *in vitro* by incrementally increasing the drug concentrations. A single colony of the parental strain was selected and cultured overnight in MHB in a shaking incubator at 37°C. An aliquot (5 ml) of the bacterial solution was transferred to 95 ml of MHB medium containing a final ciprofloxacin concentration of 0.004 μg/ml and incubated at 37°C for 12–14 h. The next day, the bacterial solution was transferred to 95 ml of MHB supplemented with an increased concentration (0.008 μg/ml) of ciprofloxacin. The procedure was then repeated to select for strains with elevated ciprofloxacin MICs of up to 4 μg/ml. At the same time, a control sample was cultured with MHB containing no drug. The final resistant strain was cultured five times in MHB with no drug and its drug sensitivity was evaluated to determine stability of the drug-resistant phenotype. The resistant strain selected *in vitro* was designated ‘TW4’.

### Antimicrobial susceptibility tests

The antimicrobial MICs were determined for the parental strain T0 and the resistant strains TN4 and TW4, using the broth microdilution method, according to the guidelines of the Clinical and Laboratory Standards Institute [11]. The following antimicrobials were tested: ciprofloxacin, ofloxacin, enrofloxacin, lomefloxacin, ceftriaxone, ceftiofur, amoxicillin, gentamicin, apramycin, amikacin, and florfenicol. *Escherichia coli* ATCC 25922 was used as the control strain.

### RNA extraction and purification

Total RNA was extracted using the RNeasy Mini Kit (Cat#74106, Qiagen, GmBH, Germany), according the manufacturer’s instructions. An RNA integrity number (RIN) was determined using an Agilent Bioanalyzer 2100 (Agilent Technologies, Santa Clara, CA, US). The total RNA was further purified using the RNeasy Micro Kit (Cat#74004, Qiagen, GmBH, Germany) and RNase-Free DNase Set (Cat#79254, Qiagen, GmBH, Germany). The RNA of each sample that passed quality control test was used for library construction.

### Library preparation and sequencing

The libraries were constructed and sequenced by Shanghai Biotechnology Corporation (No.151 Libing Road, Zhangjiang High-tech Park, Shanghai, China). The rRNA-depleted and RNA-fragmented libraries were constructed with sequential first- and second-strand cDNA synthesis, followed by end repair, adenylation of the 3' ends, ligation of adapters, and enrichment of the cDNA templates, according to experimental instructions. The quality and quantity of each libraries were determined with a Qubit® 2.0 Fluorometer and Agilent Bioanalyzer 2100, respectively. In total, three purified libraries were sequenced (2×100 bp, paired-end) using the IlluminaHiSeq2500 apparatus. The sequencing data were submitted to the National Center for Biotechnology Information Sequence Read Archive under Accession No. SRP100813.

### Data analysis

Raw reads from the sequenced libraries were subjected to quality control to filter out low-quality reads and trim the adaptor sequences. The remaining “clean reads” were mapped to the *Salmonella* Typhimurium strain LT 2 genome (National Center for Biotechnology Information reference sequence: NC_003197.1) using the Bowtie2 (version 2–2.0.5) tool [12].

The reads per kbp million mapped reads (RPKM) value for each gene was calculated for three samples and used to calculate the log(fold change) as log2(RPKM resistant strain/RPKM parental strain). The differentially expressed genes (DEGs) were detected with the DEGseq software, with a cutoff threshold of an absolute log2(fold change) >1 and a *P* value <0.001 [13].

A Gene Ontology (GO) functional enrichment analysis provided the GO terms for the DEGs that were substantially enriched relative to the genome background, to determine the biological function of the DEGs. The analysis first maps all DEGs to GO terms with the database (http://www.geneontology.org/) and the gene numbers were calculated for every term. Ultra-geometric tests are then used to identify the GO terms considerably enriched among the DEGs relative to the genomic background.

A pathway-based analysis provided further insight into the biological functions of the DEGs. The public pathway database, Kyoto Encyclopedia of Genes and Genomes (KEGG), was used to perform a pathway enrichment analysis of the DEGs. This analysis identified metabolic pathways or signal transduction pathways associated with the DEGs that were significantly enriched relative to the entire genomic background. In this study, significant pathways were identified as those with *P* values < 0.05 and the false-discovery rate (FDR) was set at < 0.05. The Q value indicates the FDR of the specific pathway, with lower values indicating greater significance.

### Validation with reverse transcription (RT)–quantitative PCR (RT–qPCR)

Significantly expressed genes were selected for validation using RT-qPCR. The RNA extracted from the three strains was converted to cDNA using the PrimeScript^TM^ RT Reagent Kit (TaKaRa Biotechnology [Dalian] Corporation, Limited). cDNA was subjected to qPCR analysis with SYBR^®^ Premix Ex Taq^TM^ II (TaKaRa) using the ViiA 7 System (Life Technologies, USA). Specific primer sets were used to evaluate the *16S rRNA*, *acrD*, *acrE*, *emrD*, *rcsC*, *soxR*, *uhpA* and *yidY* transcripts (Table 1). All reactions were performed in triplicate and the experimental data were analyzed using the 2^−ΔΔCT^ method. The gene expression levels for TN4 and TW4 were compared with those of parental strain T0 based on the cDNA sequencing results.

**Table 1:** Primers used in this study.

Gene	Primer sequence (5′→3′)*	Product length (bp)
*16S rRNA*	F: TGTAGCGGTGAAATGCGTAG	161
	R: CAAGGGCACAACCTCCAAG	
*acrD*	F: GCTACTCGCTACCTGGATGC	159
	R: TACAGCGTGGCGTCTAACAG	
*acrE*	F: CACGGTTCAACTGGTAATG	181
	R: CTGGACGCCTTCATCAAT	
*soxR*	F: AGTTAGACCGACGTATTCAT	158
	R: TTAATCATCTTCAAGCAGCC	
*yidY*	F: CTGATTGGTTGTAGCATAGTGA	95
	R: CGAGATTGGTAAGGGATTTCTT	
*emrD*	F: CCTCGTCGGCATGTCTATTT	133
	R: GAGCGTTCTCGCCATTACTC	
*uhpA*	F: GAGCGTTCTCGCCATTACTC	182
	R: GCTAAGCCAACTGCCAAAAG	
*rcsC*	F: CACGGCGATGAATAACTC	146
	R: GATAATTGGCGGTGATATGG	

* In each primer sequence, the first letter indicates the orientation: F, forward; R, reverse.

## Results

### Confirmation of colonization

**Fig 1:**
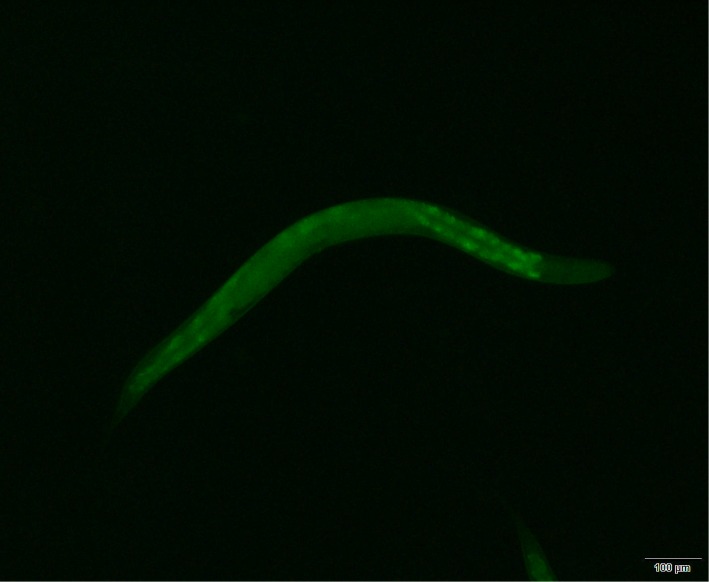
Fluorography of the colonization model (Magnification, 100×).

To confirm that *Salmonella* Typhimurium had colonized the nematodes, we constructed *Salmonella* Typhimurium expressing the *GFP* gene. After induction, we observed obvious green fluorescence in the recombinant strain and in the digestive tracts of the nematodes colonized by recombinant strain, under a fluorescence microscope (Fig 1).

### Antimicrobial susceptibility of the two strains

The MIC of ciprofloxacin for the parental strain T0 was less than 0.004 μg/ml. After selection, the MICs for the two resistant strains, TN4 and TW4, were 4 μg/ml. The MICs of 11 drugs for the two resistant strains had all increased to different degrees (Table 2). TN4 showed intermediary or resistance to FQs (ciprofloxacin, ofloxacin, and enrofloxacin) and ceftriaxone, whereas TW4 showed intermediary or resistance to FQs (ciprofloxacin, ofloxacin, enrofloxacin, and lomefloxacin) and florfenicol. The two resistant strains also showed reduced susceptibility to other drugs, but did not become resistant.

**Table 2:** Antimicrobial susceptibility of the three strains.

Strains	Fluoroquinolone MICs (μg/ml)				Other antimicrobial MICs (μg/ml)						
	CIP	OFX	ENR	LOM	CRO	CEF	AMX	GEN	APR	AMK	FLO
T0	<0.004	<0.004	<0.004	0.015	<0.25	<0.25	1	1	2	0.5	2
TN4	4	4	1	2	2	<0.25	2	2	8	1	4
TW4	4	4	2	4	1	0.5	2	1	2	0.5	16

CIP: ciprofloxacin, OFX: ofloxacin, ENR: enrofloxacin, LOM: lomefloxacin, CRO: ceftriaxone, CEF: ceftiofur, AMX: amoxicillin, GEN: gentamicin, APR: apramycin, AMK: amikacin, FLO: florfenicol.

### Differential gene expression analysis

**Fig 2:**
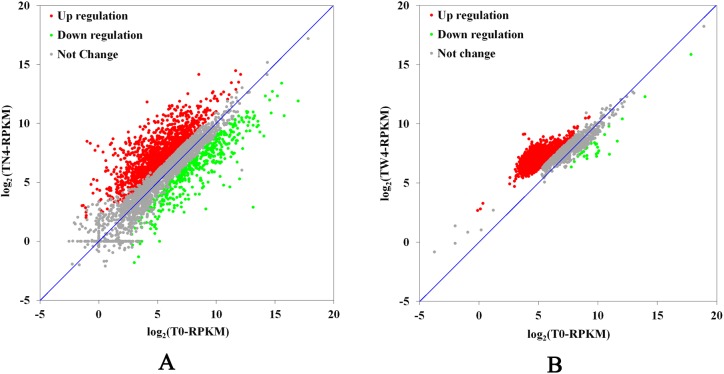
Scatterplot of the gene expression levels of the resistant and parental strains. The vertical coordinates represent the log2-transformed RPKM values for each gene in the resistant strain, and the horizontal coordinates represent the log2-transformed RPKM values for each gene in the parental strain. Green, upregulated genes; red, downregulated genes; gray, unchanged gene. A: resistant strain TN4 and parental strain T0; B: resistant strain TW4 and parental strain T0.

A comparison of TN4 and T0 showed the differential expression of 2,277 of the 3,844 genes predicted in the *Salmonella* Typhimurium genome. Of these, 1,833 genes (47.7%) were upregulated (≥ 2-fold change, *P* < 0.05) and 444 genes (11.6%) were downregulated (≥ 2-fold change, *P* < 0.05) (Fig 2A). A comparison of TW4 and T0 showed the differential expression of 3,464 of the 4,517 genes predicted in the *Salmonella* Typhimurium genome. Of these, 3,433 genes (76.0%) were upregulated (≥ 2-fold change, *P* < 0.05) and 31 genes (0.7%) were downregulated (≥ 2-fold change, *P* < 0.05) (Fig 2B). These gene expression patterns demonstrate the presence of diverse and complex regulatory systems in bacteria.

### Analysis of DEGs related to drug resistance and the two-component system (TCS)

Classification of DEGs according to their gene annotation showed that 28 DEGs in the two resistant strains were associated with drug resistance and were directly related to drug delivery systems (Table 3), including 15 genes in TN4 (13 upregulated and two downregulated) and 21 genes in TW4 (all upregulated). Eight genes were differentially expressed in both strains, with six genes showing higher expression levels in TW4, and two genes showing higher expression levels in TN4.

**Table 3:** DEGs related to drug resistance in the two resistant strains.

Gene name	Gene annotation	Up/Downregulation (resistant/parent) and fold change*	
		TN4	TW4
*aadA*	Aminoglycoside resistance protein	-	Up (5.42)
*acrA*	Acridine efflux pump	Up (4.14)	-
*acrD*	RND family aminoglycoside/multidrug efflux pump	Up (2.45)	Up (4.42)
*acrE*	Acriflavine resistance protein E	-	Up (8)
*acrR*	AcrAB operon transcriptional repressor	Down (2.22)	-
*Bcr*	Bicyclomycin/multidrug efflux system protein	Up (2.19)	Up (4.33)
*emrD*	Multidrug resistance protein D	Up (9.22)	Up (3.49)
*gyrA*	DNA gyrase, subunit A	Up (3.42)	-
*mdfA*	Multidrug translocase	Up (2.67)	-
*mdlB*	Multidrug ABC transporter permease/ATP-binding protein	Up (2.75)	-
*mdtK*	Multidrug efflux protein	-	Up (3.91)
*parC*	DNA topoisomerase IV, subunit A	Up (4.8)	-
*pmrD*	Polymyxin resistance regulatory protein PmrD	-	Up (2.84)
*rarD*	Chloramphenicol resistance	-	Up (3.82)
*soxR*	Redox-sensing transcriptional activator SoxR	Down (7.71)	-
*STM0257*	Drug efflux protein	-	Up (8.6)
*STM1619*	Cryptic aminoglycoside resistance gene	-	Up (4.84)
*STM2126*	Multidrug efflux system subunit MdtA	-	Up (3.24)
*yadG*	Multidrug ABC transporter ATPase	-	Up (5.43)
*yceE*	Drug efflux system protein MdtG	-	Up (3.09)
*yceL*	Multidrug resistance protein MdtH	-	Up (3.87)
*ydhJ*	Multidrug resistance efflux pump	-	Up (4.02)
*yegB*	Multidrug efflux system protein MdtE	Up (2.51)	Up (4.02)
*yegN*	Multidrug efflux system subunit MdtB	Up (3.45)	Up (4.24)
*yegO*	Multidrug efflux system subunit MdtC	Up (2.42)	Up (4.15)
*yhiH*	Multidrug ABC transporter ATPase	Up (2.64)	Up (3.09)
*yidY*	Multidrug efflux system protein MdtL	Up (8.12)	Up (7.11)
*yohG*	Multidrug resistance outer membrane protein MdtQ	-	Up (5.28)

* In the Up/Downregulation (resistant/parent) and fold change column, “–” means no significant difference with regard to gene expression.

Classification of the DEGs in the two resistant strains according to their gene annotation showed that 26 genes were associated with TCS (S1 Table), including 19 genes in TN4 (14 upregulated and five downregulated) and 25 genes in TW4 (all upregulated). Eighteen DEGs were expressed in both strains, five of which showed higher expression levels in TW4 and eight of which showed higher expression levels in TN4.

### GO functional enrichment analysis of DEGs

**Fig 3:**
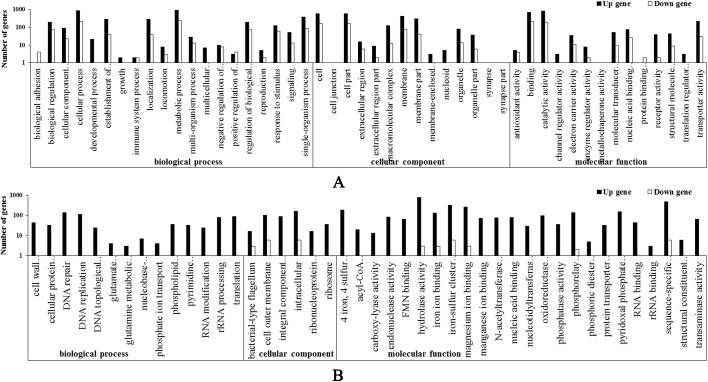
Gene Ontology terms for DEGs grouped into functional categories. The vertical and horizontal coordinates represent the number of genes and the GO functional terms, respectively. Red, upregulated genes; green, downregulated genes. A: resistant strain TN4; B: resistant strain TW4.

To further clarify the gene functions, we performed GO functional analysis of the DEGs. In TN4, 1,946 GO terms were associated with the total 3,844 *Salmonella* genes and were classified into 48 functional categories (Fig 3A). In TW4, 3,615 GO terms were associated with the total 4517 *Salmonella* genes and were classified into 44 functional categories (Fig 3B). These were numerous DEGs in the transcript libraries of the three samples, which encoded many proteins related to structure, regulation, and metabolism.

### KEGG pathway analysis

In this study, the two FQ-resistant samples of *Salmonella* Typhimurium were mapped to a reference standard to determine the significant differences in their gene enrichment pathways. In TN4, 1,601 DEGs mapped to 126 KEGG pathways, including 757 genes that were distributed in seven significantly affected metabolic pathways (Table 4). In TW4, 1,167 DEGs mapped to 114 KEGG pathways, including 252 genes distributed in seven significantly affected metabolic pathways (Table 5). These annotations of the two FQ-resistant *Salmonella* Typhimurium strains provide a valuable resource of the further investigation of the metabolic processes and functions associated with drug resistance.

**Table 4:** Pathways significantly differently enriched in resistant strain TN4 and parental strain T0.

Pathway	Differential genes	Upregulated genes	Downregulated genes	Total genes
Metabolic pathways	463	384	79	939
ABC transporters	138	119	19	245
Ribosome	63	57	6	92
Oxidative phosphorylation	46	44	2	56
Peptidoglycan biosynthesis	29	29	0	37
D-glutamine and D-glutamate metabolism	10	10	0	10
Photosynthesis	8	8	0	8

**Table 5:** Pathways significantly differently enriched in resistant strain TW4 and parental strain T0.

Pathway	Differential genes	Upregulated genes	Downregulated gene	Total genes
ABC transporters	154	153	1	176
Carbon metabolism	47	47	0	113
Glycolysis / Gluconeogenesis	15	14	1	39
Ribosome	13	13	0	78
Methane metabolism	12	12	0	30
Oxidative phosphorylation	8	8	0	43
Citrate cycle	3	3	0	26

### RT-qPCR analysis

**Fig 4:**
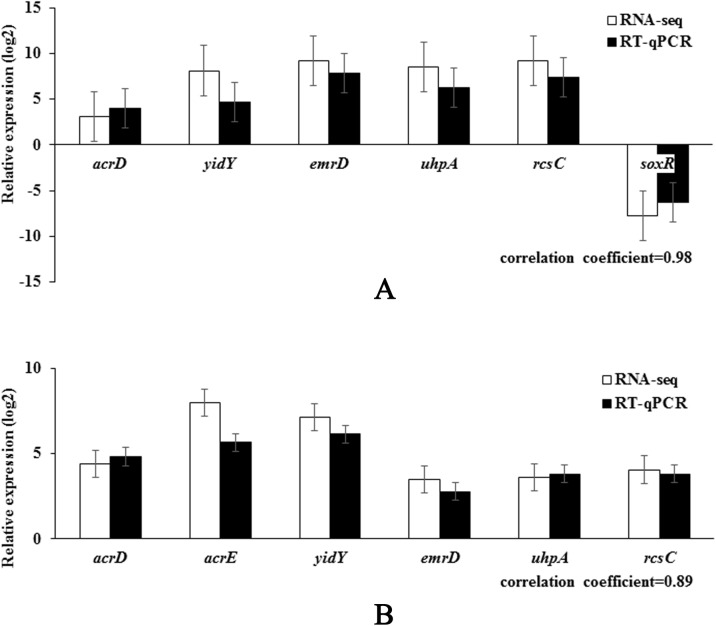
Validation of the RNA-seq assay of selected genes with a RT-qPCR analysis. RT-qPCR data confirmed the expression trends observed in the RNA-Seq data for a number of genes in resistant strains TN4 (A) and TW4 (B) compared with parental strain T0.

To verify the RNA-seq results, some of the DEGs were analyzed with RT-qPCR. The relative expression deduced with RT–qPCR was compared with the results of the RNA-seq expression analysis. As shown in Fig 4, the RT–qPCR results correlated significantly positively with the RNA-seq results (correlation coefficients 0.89–0.98, *P* < 0.05). Similar patterns of DEGs were observed in RT-qPCR and RNA-seq analyses in terms of upregulation, downregulation and fold changes in expression. In general, the RNA-seq results were confirmed by the RT–qPCR results, indicating the reliability and accuracy of the RNA-seq expression analysis.

## Discussion

Researchers generally use drugs to select drug-resistant strains *in vitro* in order to study the mechanisms of drug resistance. However, this method may be too simple to accurately reflect the mechanisms that occur *in vivo*. In this study, we report for the first time the use of a *C*. *elegans*–*Salmonella* Typhimurium infection model to select a drug-resistant strain *in vivo* for a transcriptome sequencing analysis. Compared with the parental strain, the two resistant strains showed significant changes in their drug-resistant phenotypes, gene expression levels, and metabolic pathways after selection.

Bacterial resistance to FQ is usually mediated by mutations in the bacterial DNA gyrase and topoisomerase IV genes, or by the overexpression of efflux system genes [14, 15]. The expression levels of these resistance-related genes directly affect the drug-resistant phenotype. In our antibiotic sensitivity tests (Table 2), the two resistant strains showed resistance to FQs and reduced susceptibility to other drugs, although this compromised susceptibility did not become resistance. Our results are consistent with the findings of Chen *et al*. [16], who reported a laboratory-selected ciprofloxacin-resistant strain that displayed resistance or reduced susceptibility to other FQs, and reduced susceptibility to some cephalosporins. They also showed that mutations in the DNA gyrase and topoisomerase IV genes, especially *gyrA* and *parC*, play an important role in resistance to FQ, and exerted synergistic effects on the efflux pumps in the laboratory-selected resistant strain [16]. In TN4, the expression of *gyrA* and *parC* increased significantly, perhaps caused by a large number of substrates (drug) combine with protein loci. Although no changes in the expression of these genes were detected in TW4, this does not preclude the generation of mutations in those genes, and this possibility requires further investigation.

Among the many resistance-related genes (Table 3), the expression of *aadA*, *STM1619*, and *pmrD*, which are associated with aminoglycoside resistance, was significantly upregulated in TW4, but was unchanged in TN4. However, there were no significant changes in the MICs of aminoglycoside, suggesting that changes in the expression of these genes may not be the main cause of bacterial resistance to aminoglycoside. The two resistant strains did not appear to be resistant to cephalosporin, suggesting that the mechanism of cephalosporin resistance is quite different from that of FQ resistance.

The other differentially expressed resistance-related genes were all efflux system genes, with 13 and 18 identified in TN4 and TW4, respectively (Table 3). Efflux pumps are very important in bacterial resistance to many classes of antimicrobial agents, especially AcrAB, AcrD, AcrEF, and MdtABC in *Salmonella* species. AcrAB–TolC is the most important efflux pump [17], and the overexpression of AcrAB–TolC is mediated by the transcriptional activators MarA and SoxS, which are regulated by the MarR and SoxR proteins, respectively [18–20]. AcrAB is also regulated by the local repressor AcrR, which inhibits the transcription of *acrA* and *acrB*, and the mutation of *acrR* contributes to the overexpression of AcrAB and increases bacterial resistance to multiple drugs [21, 22]. No significant increase in the expression of *acrA* or *acrB* was observed in TW4, whereas the expression of *acrD*, *acre*, and other efflux-pump-encoding genes increased significantly in TN4. In TN4, a significant increase in the expression of *acrA* and *acrD* was observed, whereas the expression of *acrR* and *soxR* was significantly reduced (Table 3). An analysis by Chen *et al*. showed that the expression of efflux pump genes *acrA*, *acrB*, *acrE*, *acrF*, *emrB*, *emrD*, and *mdlB* was substantially increased (> 2-fold) among FQ-resistant mutants [17]. Smith *et al*. also reported that when a hypersensitive *E*. *coli* carried the *emrD* gene, the gene conferred enhanced resistance to several antimicrobials [23]. In the two resistant strains, the expression of several genes encoding these efflux pumps was significantly increased. Our results suggest that the overexpression of efflux pumps was one of the main mechanisms underlying FQ resistance in these two resistant strains. More DEGs encoded efflux pumps in TW4 than in TN4 and the expression of most the same differentially expressed efflux pump genes was higher in TW4 than in TN4, whereas the expression of the efflux pump repressor gene and the mar operon was downregulated in TN4, but not in TW4. It is possible that other unknown or nondifferentially expressed genes associated with the efflux pump repressor and the mar operon regulate the expression of efflux pumps in TW4, although this speculation requires further investigation.

Many bacteria have large numbers of TCSs, which are composed of histidine kinase and a response regulator protein, and play an important role in bacterial drug resistance. OmpR/Envz mediates an osmotic-stress-related adjustment of TCS, by changing the OmpR phosphorylation status at the transcriptional level, regulating the expression of the outer membrane protein OmpF/C. EvgA can also combine with the promoter regions of the efflux pump genes *yhiU* and *emrK* to regulate gene expression [24]. In *Salmonella* Typhimurium, CpxAR and BaeSR influence the outer membrane proteins STM1530 and OmpD, leading to the development of ceftriaxone resistance [25]. The BaeSR system also increases multidrug resistance and metal resistance by inducing the AcrD and MdtABC drug efflux systems [26]. TCSs can influence the transcription and expression of the relevant drug-resistant genes through self-regulation and their interactions with other systems, by identifying a series of host environments, regulating bacterial adaptability, and resisting adverse environmental conditions. There have been few reports of the relationship between TCSs and drug resistance in *Salmonella*, and further study of the TCS will extend our understanding of the resistance mechanisms in *Salmonella*.

We also observed an interesting phenomenon in the gene expression patterns of *Salmonella* Typhimurium. Of the genes differentially expressed between the two resistant strains, more genes were downregulated in TN4, whereas more genes were upregulated in TW4, in which almost no genes were downregulated (accounting for only 0.9% of the total number of DEGs). The fold change in the gene expression levels in TW4 were more dispersed than those in TN4, whereas those in TW4 was more concentrated (Fig 2). When the DEGs were grouped into GO functional categories (biological process, cellular component, and molecular function), the TN4 DEGs appeared to be spread relatively evenly across the three categories, whereas most of the TW4 DEGs were concentrated in molecular function (Fig 3). The DEGs were significantly associated with seven different metabolic pathways, with the “ABC transporters”, “ribosome” and “oxidative phosphorylation” pathways identified in both resistant strains (Tables 4 and 5). Bacteria switch from drug sensitivity to drug resistance in many ways, utilizing energy production, protein synthesis, substance transport, and so on. The changes in the common metabolic pathways of the two resistant strains provided the basis for these different changes, and these different metabolic pathways may be where the differences between the two resistant strains can be identified. The resistant strain selected *in vitro* need only combat the drug in the growth environment, whereas the strain selected *in vivo* must also resist the host’s immune system in the complex environment of the worm’s body. The differences in gene expression levels showed that the *in vitro*-selected changes may have occurred predominantly in resistance-related genes while the expression of the basic metabolic genes was maintained. However, in the *in vivo*-selected strain, the changes may have occurred in the genes of the overall bacterial systems, including in immune- and self-protection-related genes, furthermore the changes in gene expression also affected metabolic pathways. This suggests that changes in drug resistance based on gene expression patterns can influence a variety of metabolic pathways, and the same three pathways were affected in the two resistant strains. However, given the currently available information, the association between resistance mechanisms and metabolic pathways, especially in *in vivo*-selected strains, requires further exploration.

To the best of our knowledge, this is the first report describing an analysis of the *in vivo* and *in vitro* selection of drug-resistant strains of *Salmonella* Typhimurium based on RNA-seq. In summary, drug resistance in *Salmonella* Typhimurium is not isolated, and involves the interaction of several resistance mechanisms. A systematic and in-depth investigation of the molecular mechanism of drug resistance in *Salmonella* Typhimurium will allow appropriate strategies to be developed to effectively control and reduce the incidence this extremely complex phenomenon.

## Supporting information

S1 Table DEGs related to the two-component system in the two resistant strains. (XLSX)
